# Supplementary material for: Free Fatty Acids from Cow Urine DMSO Fraction Induce Cell Death in Breast Cancer Cells without Affecting Normal GMSCs
Source: Biomedicines. 2023 Mar 13;11(3):889. doi: 10.3390/biomedicines11030889 (PMC10046047; doi:10.3390/biomedicines11030889)
Supplement: Supplementary file 1 [file biomedicines-11-00889-s001.zip › biomedicines-2233765-supplementary.pdf]

Supplementary Data

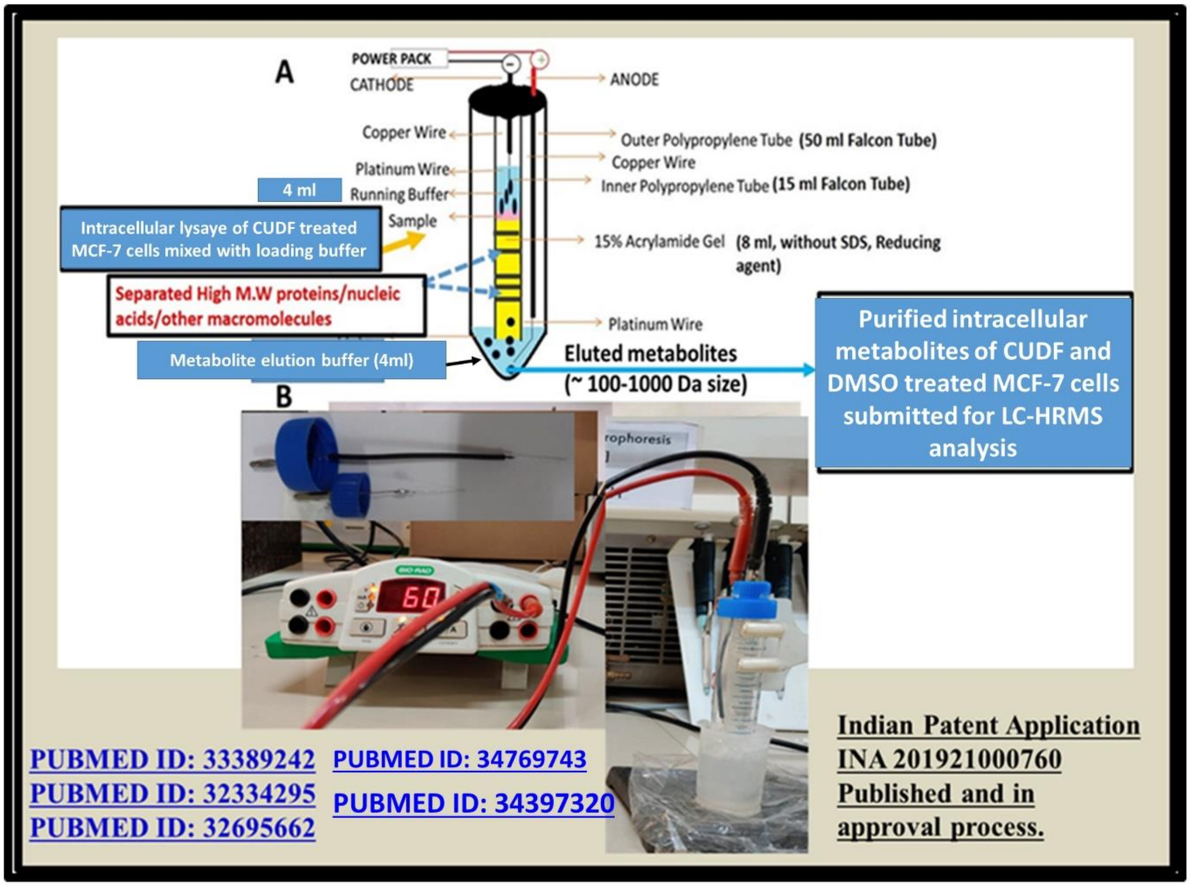

Figure S1. A flow diagram of novel and in-house developed vertical tube gel electrophoresis (VTGE) system for the purification of intracellular metabolites.

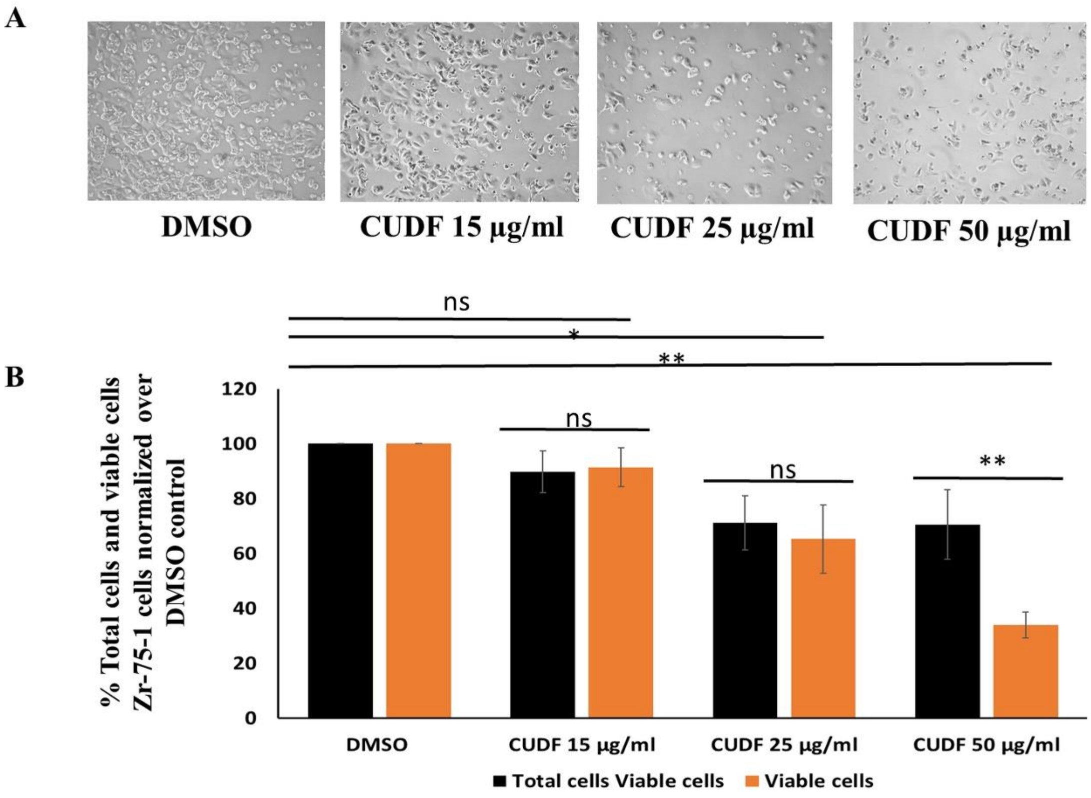

Figure S2. Cow urine DMSO fraction (CUDF) shows significant reduction in the viability of ZR-75-1 breast cancer cells.

(A) ZR-75-1 cancer cells were treated by DMSO and varied concentration of CUDF (15 µg/ml, 25 µg/ml and 50 µg/ml) for 72 hr. Routine microscopy was performed at 100X to observe the cell number and cellular morphology. (B) MCF-7 cancer cells were treated by DMSO and CUDF (50 µg/ml) for 72 hr. Percentage total and cell viability of MCF-7 cancer cells estimated by Trypan blue dye exclusion assay and normalized over DMSO control. Data are represented as mean ± SD. Each experiment was conducted independently three times. The bar graph without an asterisk denotes that there is no significant difference compared to DMSO control. * Significantly different from DMSO control at the P-value ≤ 0.05. ** Significantly different from DMSO control at P-value ≤ 0.01. *** Significantly different from DMSO control at P-value ≤ 0.001.

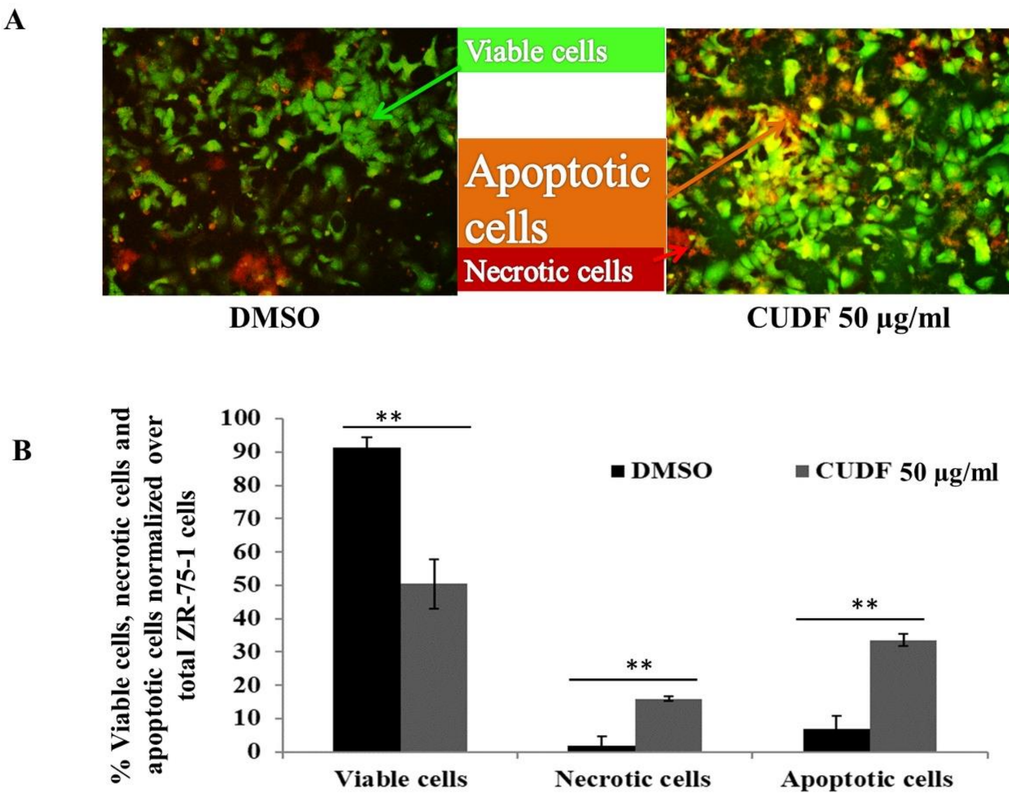

Figure S3. Cow urine DMSO fraction (CUDF) induces apoptosis in ZR-75-1 cancer cells by dual acridine orange (AO)/Ethidium bromide (EB) staining.

(A) ZR-75-1 breast cancer cells were treated by DMSO and CUDF (50 µg/ml) for 72 hr. At the end of incubation, cells were harvested and processed for acridine orange (AO)/Ethidium bromide (EB) staining as described under "experimental procedure". The fluorescent microscopy images were captured at 40X magnification. (B) Percentage viable cells, necrotic cells and apoptotic cells in ZR-75-1 cancer cells were estimated by counting of cells differentially stained with AO/EB. Green color (normal cells). Yellow-green fluorescence by acridine orange (AO) Early apoptotic cells): Crescent or granular group of cells shows (Late apoptotic cells): EB stained orange fluorescence (Necrotic cells). Data are represented as mean ± SD. Each experiment was conducted independently three times. The bar graph without an asterisk denotes that there is no significant difference compared to DMSO control. * Significantly different from DMSO control at the P-value ≤ 0.05. ** Significantly different from DMSO control at P-value ≤ 0.001. *** Significantly different from DMSO control at P-value ≤ 0.001.

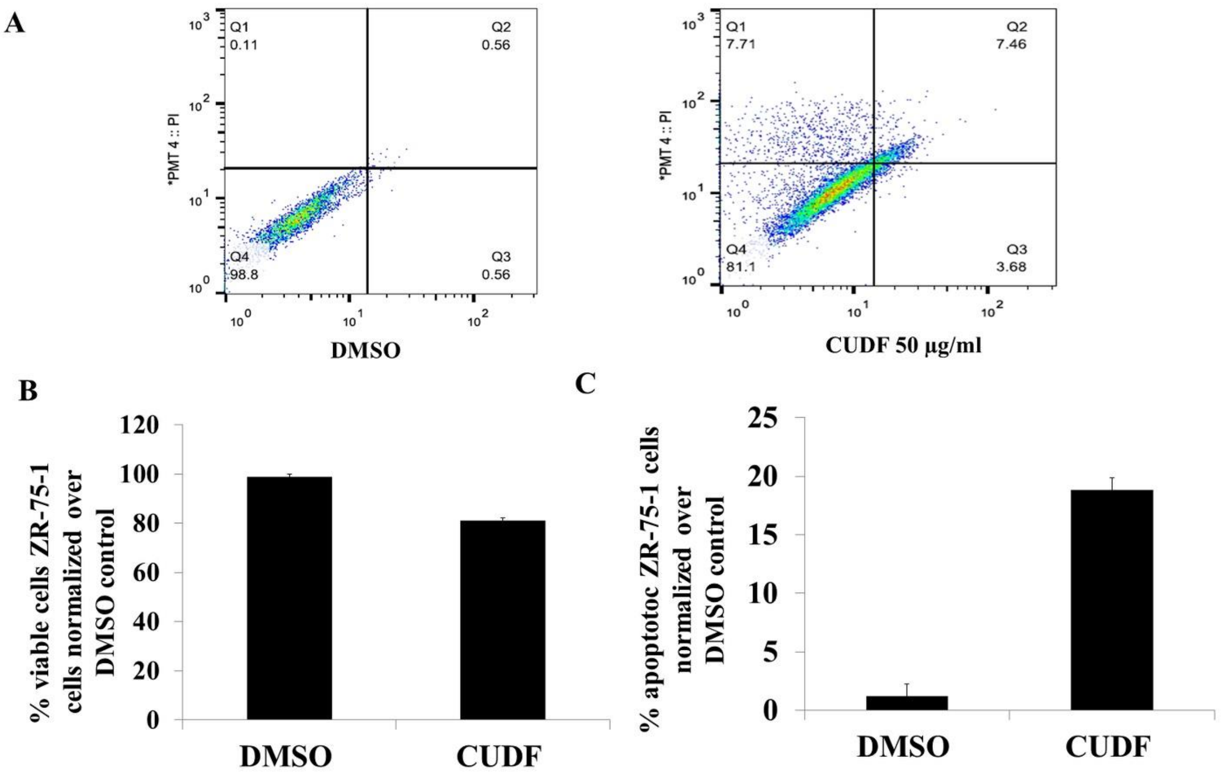

Figure S4. Cow urine DMSO fraction (CUDF) treatment upon ZR-75-1 breast cancer cells promotes apoptotic cell death.

(A) ZR-75-1 cancer cells were treated by DMSO and CUDF (50 µg/ml) for 72 hr. At the end of treatment, harvested ZR-75-1 cells were subjected to PI/annexin V staining and analyzed by flow cytometer. The scatter plots of cells were stained with PI and Annexin V conjugated with FITC for the analysis of apoptotic cells in ZR-75-1. (B) ZR-75-1 cancer cells were treated by DMSO and CUDF (50 µg/ml) for 72 hr. At the end of treatment, harvested ZR-75-1 cells were subjected to PI/annexin V staining and analyzed by flow cytometer. Percentage of cell viability was calculated from number of PI and Annexin V negative cells in the quadrant 4 of scattered plot divided by total number of cell. (C) ZR-75-1 cancer cells were treated by DMSO and CUDF (50 µg/ml) for 72 hr. At the end of treatment, harvested MCF-7 cells were subjected to PI/annexin V staining and analyzed by flow cytometer. Percentage of apoptotic cell was calculated from number of cells in the quadrant 3 and 4 stained with Annexin V and PI divided by the total number of cell. Data are represented as mean ± SD. Each experiment was conducted independently three times. The bar graph without an asterisk denotes that there is no significant difference compared to DMSO control. * Significantly different from DMSO control at the P-value ≤ 0.05. ** Significantly different from DMSO control at P-value ≤ 0.001. *** Significantly different from DMSO control at P-value ≤ 0.001.

Compound Label	Name	*m/z*	RT	Algorithm	Mass
Tetracosanedioic acid	**Tetracosanedioic acid**	399.3537	14.871	Auto MS/MS	398.3465

MS Spectrum

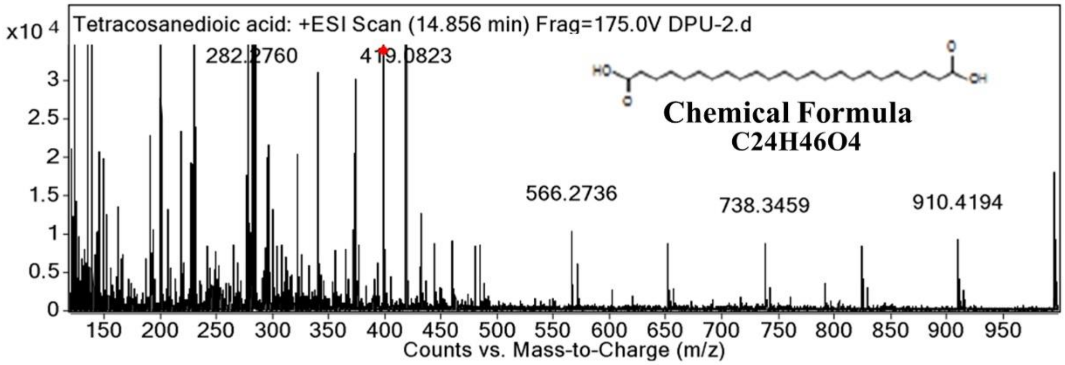

MS Spectrum Peak List

m/z	z	Abund
124.0851		47872.12
136.0623		54191.03
139.0482		51758.3
200.0918		56061.94
230.1376		39087
282.276	1	319787.27
283.2793	1	71075.13
284.3276	1	94321.62
418.0749		57135.61
419.0823	1	90879.39

Figure. S5. LC-HRMS fragmentation mass ion spectra of tetracosanedioic acid
MCF-7 cancer cells were treated with CUDF. Intracellular lysate of MCF-7 cells were prepared by hypotonically and processed for the purification of metabolite by VTGE. Then VTGE purified intracellular metabolite was submitted to LC-HRMS. A detailed procedure on LC-HRMS analysis is given in the experimental procedure.

Compound Label	Name	*m/z*	RT	Algorithm	Mass
13(Z)-Docosenoic acid	**13(Z)-Docosenoic acid**	343.2923	10.898	Auto MS/MS	338.3135

MS Spectrum

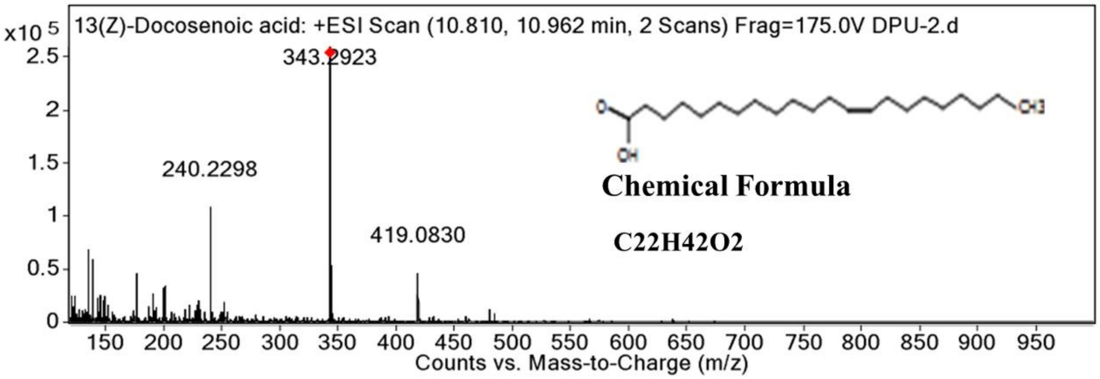

MS Spectrum Peak List

m/z	z	Abund
136.0625	2	69621.88
139.0483	1	62810.35
177.052		28881.79
177.0672	1	45553.57
191.0125		29525.33
201.0996	1	34687.09
240.2298	1	108093.58
343.2923	1	267578.71
344.2952	1	54010.93
419.083	1	47718.38

Figure. S6. LC-HRMS fragmentation mass ion spectra of 13(Z)-Docosenoic acid.
MCF-7 cancer cells were treated with CUDF. Intracellular lysate of MCF-7 cells were prepared by hypotonically and processed for the purification of metabolite by VTGE. Then VTGE purified intracellular metabolite was submitted to LC-HRMS. A detailed procedure on LC-HRMS analysis is given in the experimental procedure.

Compound Label	Name	*m/z*	RT	Algorithm	Mass
Nervonic acid	**Nervonic acid**	371.3226	12.835	Auto MS/MS	366.3439

MS Spectrum

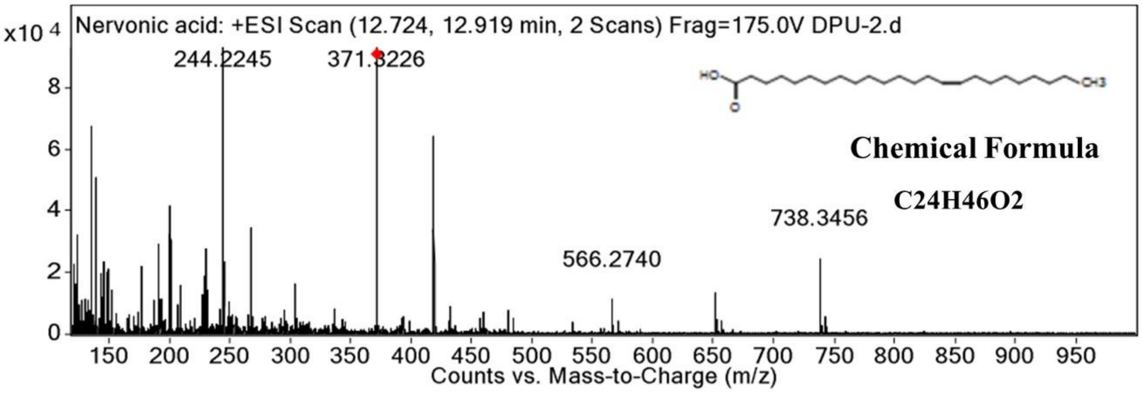

MS Spectrum Peak List

m/z	z	Abund
124.0851		33164.58
136.0622	2	68072.22
139.0482	1	53963.69
191.012		30698.31
200.0919		42706.18
201.099		33664.11
244.2245	1	139475.56
268.2602	1	35868.97
371.3226	1	93522.18
419.0823	1	65773.71

Figure. S7. LC-HRMS fragmentation mass ion spectra of nervonic acid.
MCF-7 cancer cells were treated with CUDF. Intracellular lysate of MCF-7 cells were prepared by hypotonically and processed for the purification of metabolite by VTGE. Then VTGE purified intracellular metabolite was submitted to LC-HRMS. A detailed procedure on LC-HRMS analysis is given in the experimental procedure.

Compound Label	Name	*m/z*	RT	Algorithm	Mass
3-hydroxy-tridecanoic acid	**3-hydroxy-tridecanoic acid**	235.1664	14.322	Auto MS/MS	230.1877

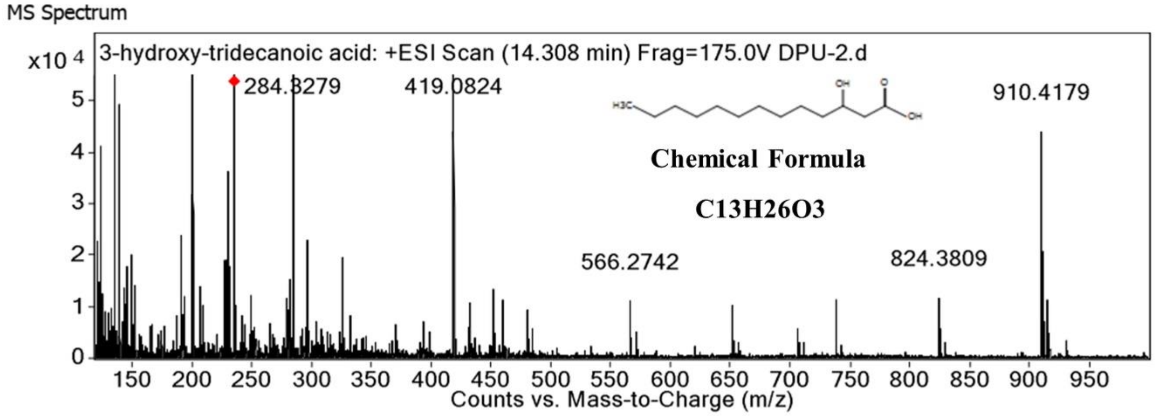

MS Spectrum Peak List

m/z	z	Abund
124.085	1	42940.22
136.0623		65038.8
139.048		51784.62
200.0918		56141.2
230.1371		36396.9
235.1664	1	55267.61
284.3279	1	68775.03
418.075		55309.28
419.0824	1	83885.64

Figure S8. LC-HRMS fragmentation mass ion spectra of 3-hydroxytridecanoic acid.
MCF-7 cancer cells were treated with CUDF. Intracellular lysate of MCF-7 cells were prepared by hypotonically and processed for the purification of metabolite by VTGE. Then VTGE purified intracellular metabolite was submitted to LC-HRMS. A detailed procedure on LC-HRMS analysis is given in the experimental procedure.

3-Hydroxycapric acid	**3-Hydroxycapric acid**	193.1158	0.89	Auto MS/MS	188.137

MS Spectrum

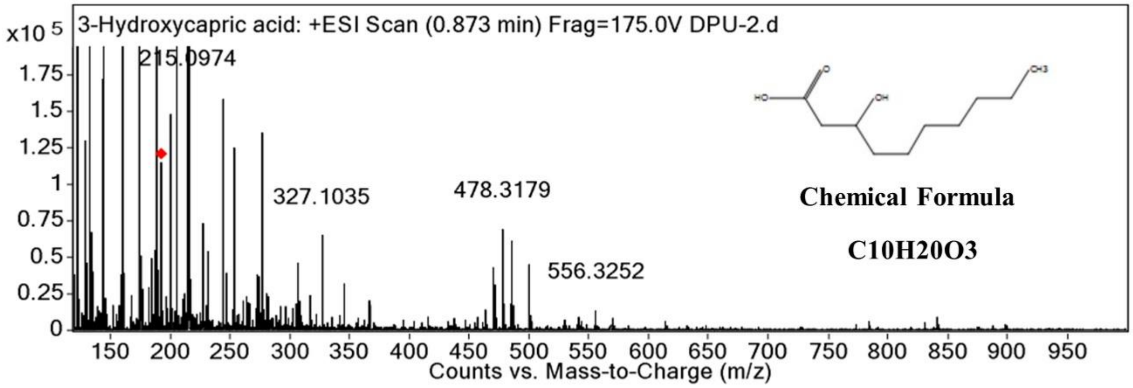

MS Spectrum Peak List

m/z	z	Abund
122.0793	1	3214158.66
133.1319	1	375042.46
144.0609	1	2062290.97
160.1423	1	416120.43
174.1576	1	553190.43
188.1732	1	200743.49
206.0311		200210.84
215.0974	1	2920364.33
216.101	1	238359.62
244.1238	1	172911.19

Figure S9. LC-HRMS fragmentation mass ion spectra of 3-hydroxycapric acid.
MCF-7 cancer cells were treated with CUDF. Intracellular lysate of MCF-7 cells were prepared by hypotonically and processed for the purification of metabolite by VTGE. Then VTGE purified intracellular metabolite was submitted to LC-HRMS. A detailed procedure on LC-HRMS analysis is given in the experimental procedure.

Tetracosanedioic acid

A

Quer	Liver Toxicity		Metabolism	Cyp Inhibitors for					Membrane Transporters			Others			
	DILI	Cyto-toxic	HLM	1A2	3A4	2D6	2C9	2C19	BBB	P-gp Inhib	P-gp Subs	hERG Bloc	MMP	AMES	MRTD (mg/c
	No	No	Yes	No	No	No	No	No	No	No	No	No	No	No	15601

Trichostatin A (TSA)

B

Quer	Liver Toxicity		Metabolism	Cyp Inhibitors for					Membrane Transporters			Others			
	DILI	Cyto-toxic	HLM	1A2	3A4	2D6	2C9	2C19	BBB	P-gp Inhib	P-gp Subs	hERG Bloc	MMP	AMES	MRTD (mg/c
	No	No	Yes	No	No	No	No	No	Yes	Yes	Yes	No	Yes	Yes	150

Figure S10 Intracellular FFA tetracosanedioic acid is predicted as a safer drug candidate over Trichostatin A (TSA), a known inhibitor of HDAC by vNN-ADMET.

Here, intracellular FFA tetracosanedioic acid and Trichostatin A (TSA) were submitted to vNN-ADMET server for the prediction of carcinogenicity, liver toxicity, substrate of P-gp and recommended therapeutic dose (RMTD).

Table S1: Molecular docking based screening of selected FFAs against several pro-tumor proteins. These selected FFAs are detected in the intracellular compartment of MCF-7 breast cancer cells treated with FFAs enriched CUDF.

**Name of ligands**	**Proteins and their PDB IDs**	**Binding energy (-kcal/mol)**	**RMSD value l.b.**	**RMSD value u.b**
**13-Docosenoic acid ($C_{22}H_{42}O_2$)** (PubChem CID 8216)	2AST (Skp1-Skp2-Cks1 in complex with a p27)	-4.0	0.000	0.000
	5L2W (CDK2/Cyclin E)	-4.8	0.000	0.000
	2W96 (CDK4 in complex with a D-type cyclin)	-5.0	0.000	0.000
	4JSN (mTORdeltaN-mLST8 complex)	-4.5	0.000	0.000
	1FOS (C-FOS:C-JUN:DNA)	-5.3	0.000	0.000
	1LFD (RAS PROTEIN)	-4.0	0.000	0.000
	4WXX (DNMT1(351-1600)	-4.4	0.000	0.000
	5A9D (Extracellular domain of PepT1)	-4.8	0.000	0.000
	5ZTY (G protein coupled receptor)	-3.1	0.000	0.000
	6OS9 (Human Neurotensin Receptor (C state)	-4.4	0.000	0.000
	4DAJ (Muscarinic Acetylcholine Receptor)	-4.2	0.000	0.000
	1C3R **(**HDAC Homolog Complexed)	-7.5	0.000	0.000
	2AST (Skp1-Skp2-Cks1 in complex with a p27)	-3.9	0.000	0.000
	5L2W (CDK2/Cyclin E)	-4.2	0.000	0.000
	2W96 (CDK4 in complex with a D-type cyclin)	-4.3	0.000	0.000
	4JSN (mTORdeltaN-mLST8 complex)	-4.6	0.000	0.000

Tetracosanedioic acid ($C_{24}H_{46}O_4$) (PubChem CID2724554)	1FOS (C-FOS:C-JUN:DNA)	-5.5	0.000	0.000
	1LFD (RAS PROTEIN)	-4.5	0.000	0.000
	4WXX (DNMT1(351-1600)	-4.1	0.000	0.000
	5A9D (Extracellular domain of PepT1)	-3.9	0.000	0.000
	5ZTY (G protein coupled receptor)	-3.4	0.000	0.000
	6OS9 (Human Neurotensin Receptor (C state)	-4.2	0.000	0.000
	4DAJ (Muscarinic Acetylcholine Receptor)	-4.5	0.000	0.000
	1C3R **(**HDAC Homolog Complexed)	-7.0	0.000	0.000
3-Hydroxytridecanoi acid ($C_{13}H_{26}O_3$) (PubChem CID: 5312749)	2AST (Skp1-Skp2-Cks1 in complex with a p27)	-4.0	0.000	0.000
	5L2W (CDK2/Cyclin E)	-4.0	0.000	0.000
	2W96 (CDK4 in complex with a D-type cyclin)	-4.8	0.000	0.000
	4JSN (mTORdeltaN-mLST8 complex)	-5.0	0.000	0.000
	1FOS (C-FOS:C-JUN:DNA)	-4.5	0.000	0.000
	1LFD (RAS PROTEIN)	-5.3	0.000	0.000
	4WXX (DNMT1(351-1600)	-4.0	0.000	0.000
	5A9D (Extracellular domain of PepT1)	-4.4	0.000	0.000
	5ZTY (G protein coupled receptor)	-4.8	0.000	0.000
	6OS9 (Human Neurotensin Receptor (C state)	-3.5	0.000	0.000

	4DAJ (Muscarinic Acetylcholine Receptor)	-4.4	0.000	0.000
	1C3R **(**HDAC Homolog Complexed)	-6.6	0.000	0.000
**Nervonic acid ($C_{24}H_{46}O_2$)** (PubChem CID 5281120)	5L2W (CDK2/Cyclin E)	-4.4	0.000	0.000
	2W96 (CDK4 in complex with a D-type cyclin)	-4.3	0.000	0.000
	4JSN (mTORdeltaN-mLST8 complex)	-4.6	0.000	0.000
	1FOS (C-FOS:C-JUN:DNA)	-4.9	0.000	0.000
	1LFD (RAS PROTEIN)	-4.9	0.000	0.000
	4WXX (DNMT1(351-1600)	-5.0	0.000	0.000
	5A9D (Extracellular domain of PepT1)	-4.4	0.000	0.000
	5ZTY (G protein coupled receptor)	-3.9	0.000	0.000
	6OS9 (Human Neurotensin Receptor (C state)	-4.1	0.000	0.000
	4DAJ(muscarinic Acetylcholine Receptor)	-4.6	0.000	0.000
	1C3R **(**HDAC Homolog Complexed)	-6.2	0.000	0.000
	2AST (Skp1-Skp2-Cks1 in complex with a p27)		0.000	0.000
	1LFD (RAS PROTEIN)	-5.6	0.000	0.000
	4WXX (DNMT1(351-1600)	-5.1	0.000	0.000
	5A9D (Extracellular domain of PepT1)	-4.3	0.000	0.000
	5ZTY (G protein coupled receptor)	-3.5	0.000	0.000

	6OS9 (Human Neurotensin Receptor (C state)	-3.9	0.000	0.000
	4DAJ(muscarinic Acetylcholine Receptor)	-3.5	0.000	0.000
	5TD7 (Histone deacetylase 10)	-4.2	0.000	0.000
	4BKX (HDAC1 complex)	-5.2	0.000	0.000
	1C3R **(**HDAC Homolog Complexed)	-6.5	0.000	0.000
3-Hydroxycapric acid ($C_{10}H_{20}O_3$) (PubChemCID26612)	2AST (Skp1-Skp2-Cks1 in complex with a p27)	-4.9	0.000	0.000
	5L2W (CDK2/Cyclin E)	-4.5	0.000	0.000
	2W96 (CDK4 in complex with a D-type cyclin)	-3.8	0.000	0.000
	4JSN (mTORdeltaN-mLST8 complex)	-4.6	0.000	0.000
	1FOS (C-FOS:C-JUN:DNA)	-5.6	0.000	0.000
	1LFD (RAS PROTEIN)	-5.1	0.000	0.000
	4WXX (DNMT1(351-1600)	-4.3	0.000	0.000
	5A9D (Extracellular domain of PepT1)	-3.5	0.000	0.000
	5ZTY (G protein coupled receptor)	-3.9	0.000	0.000
	6OS9 (Human Neurotensin Receptor (C state)	-3.5	0.000	0.000
	4DAJ(Muscarinic Acetylcholine Receptor)	-4.2	0.000	0.000
	1C3R **(**HDAC Homolog Complexed)	-6.1	0.000	0.000
